# Case Report: Management of a challenging case presenting with dysphagia after thyroidectomy requiring administration of liquid ^131^I via MIC-KEY low-profile tube—a multidisciplinary approach

**DOI:** 10.3389/fmed.2026.1737911

**Published:** 2026-01-29

**Authors:** Ahmed Ebada Salem, Sandra Ramirez, Brian R. Weston, Akram Hussein, Simone Krebs

**Affiliations:** 1 Department of Nuclear Medicine, The University of Texas MD Anderson Cancer Center, Houston, TX, United States; 2 Department of Radiodiagnosis and Intervention, Alexandria University, Alexandria, Egypt; 3 Department of Environmental Health & Safety, Sustainability and Emergency Management, The University of Texas MD Anderson Cancer Center, Houston, TX, United States; 4 Department of Gastroenterology, Hepatology and Nutrition, The University of Texas MD Anderson Cancer Center, Houston, TX, United States; 5 Departmnt of Imaging Physics, The University of Texas MD Anderson Cancer Center, Houston, TX, United States

**Keywords:** dysphagia, liquid ^131^I-131, MIC-KEY low-profile gastrostomy feeding tube, papillary thyroid cancer, total thyroidectomy

## Abstract

Radioactive iodine (RAI) therapy with 131-iodine (^131^I) remains a cornerstone in the postoperative management of differentiated thyroid carcinoma (DTC). The standard route of administration is oral, typically in capsule form and less commonly as a liquid. However, in patients with severe dysphagia and compromised airway, traditional oral ^131^I delivery may be contraindicated due to the risk of aspiration and radioactivity contamination. These scenarios present unique clinical challenges that necessitate deviation from conventional protocols. While liquid ^131^I may be administered through the mouth or via nasogastric or percutaneous gastrostomy tubes, there is currently no standardized approach, and potential risks, such as radiotracer spillage or retention at the tube site, must be considered. We present the case of a 37-year-old woman with BRAF V600E-mutated papillary thyroid carcinoma (PTC) who developed persistent dysphagia following total thyroidectomy as a result of a postoperative surgical complication. She required long-term enteral feeding via a gastrostomy tube and was referred for pretherapy evaluation and assessment of the feasibility of therapeutic ^131^I administration in the setting of a higher risk of recurrence. Following comprehensive coordination among nuclear medicine, endocrinology, gastroenterology, radiopharmacy, and radiation safety teams, a multidisciplinary decision was made to administer liquid ^131^I via a low-profile MIC-KEY gastrostomy tube under stringent radiation safety protocols. Notably, no significant radioactivity was spilled or retained at the tube site, and administration was completed. Given the paucity of literature and lack of evidence-based guidelines to address such complex clinical scenarios, this report demonstrates the feasibility and safety of administering liquid ^131^I via a low-profile MIC-KEY gastrostomy tube and underscores the importance of individualized treatment planning and effective multidisciplinary collaboration.

## Introduction

The incidence of thyroid cancer (TC) has increased significantly over recent decades, largely attributed to improved detection through widespread imaging and more frequent tissue sampling. Thyroid cancer is currently the ninth most common malignancy worldwide and the leading cancer diagnosis among adolescents and adults aged <40, with a higher prevalence in women. Papillary thyroid carcinoma (PTC) accounts for more than 80% of all thyroid cancers and, along with follicular and oncocytic variants, is categorized as differentiated thyroid carcinoma (DTC) (1).

The American Joint Committee on Cancer (AJCC 8th edition) TNM staging system incorporates several key prognostic factors: patient age (with individuals under 55 years typically demonstrating a more favorable prognosis), tumor size and local invasion (T), presence and laterality of regional lymph node involvement (N), and distant metastasis (M), most commonly involving the lungs and bones (2). Risk stratification is essential for guiding management decisions. The 2025 updated American Thyroid Association (ATA) guidelines modernize this process by integrating histopathologic features with early postoperative thyroglobulin levels and preoperative imaging findings. This dynamic framework supports more precise and individualized recommendations for adjuvant radioactive iodine (RAI) therapy, further reducing routine RAI use in low-risk patients. Intermediate-risk disease is currently subdivided into low-intermediate and intermediate-high categories to better reflect true recurrence risk and enhance treatment personalization, while high-risk disease continues to warrant adjuvant RAI therapy. The updated guidelines also emphasize early response-to-therapy assessment to refine surveillance, inform selective treatment strategies, and guide tailored long-term follow-up (3). Nevertheless, total thyroidectomy, with or without adjuvant radioactive iodine (RAI) therapy using 131-iodine (^131^I), remains the cornerstone of treatment for the majority of patients with DTC (1, 4). ^131^I has a physical half-life of approximately 8 days and emits both beta particles and gamma rays. The beta emissions deliver targeted cytotoxic effects to residual thyroid tissue or metastatic disease, while the gamma emissions facilitate imaging. This dual functionality underlies its role in both the diagnostic and therapeutic management of DTC (5). RAI has been used in the treatment of DTC for nearly a century, originating from the pioneering research of Dr. Saul Hertz (6). Postoperative RAI has demonstrated a survival benefit in high-risk patients, although its utility in low- and intermediate-risk populations remains an area of ongoing research. The therapeutic goals of RAI include the following: (i) ablation of remnant thyroid tissue to facilitate thyroglobulin-based surveillance, (ii) eradication of microscopic disease to reduce the risk of recurrence, and (iii) treatment of known metastases. Its administration requires strict radiation safety protocols to prevent contamination and minimize radiation exposure to healthcare providers, caregivers, and the public (3).

RAI is typically administered orally, either in capsule form or, less commonly, as a liquid preparation (7). Several alternative administration protocols have been explored to address challenges in patients who cannot tolerate standard oral ^131^I delivery. These approaches include the use of liquid ^131^I via nasogastric or gastrostomy tubes, reformulation as iodine–gelatin complexes to reduce gastrointestinal mucosal exposure, endoscopic placement of capsules, and, in select cases, parenteral administration (8). This report presents a unique case involving a patient with severe dysphagia who was unable to swallow ^131^I capsules or ingest the liquid formulation. Management of this case required careful coordination among a multidisciplinary team to ensure safe and effective delivery of ^131^I through an enteric route. Given the paucity of literature and lack of evidence-based guidelines addressing such clinical scenarios, this case underscores the feasibility and safety of administering liquid ^131^I via a low-profile MIC-KEY gastrostomy tube under strict radiation safety protocols. Successful execution of this approach requires close collaboration among nuclear medicine, gastroenterology, radiation safety, and radiopharmacy teams to ensure accurate dosing, minimize contamination risk, and prevent radioiodine spillage.

## Case presentation

**Figure 1:**
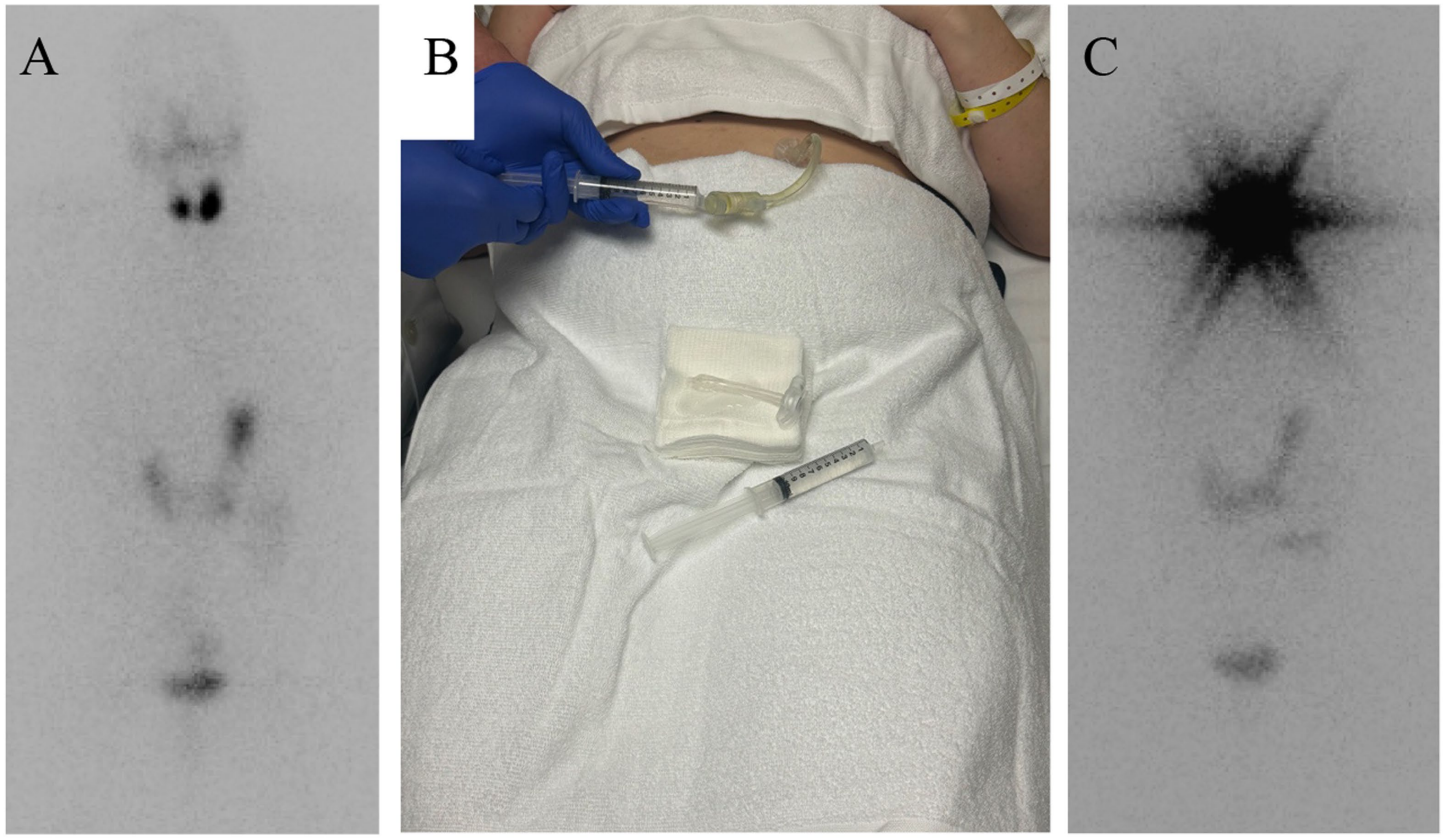
**(A)** Pre-therapy whole-body scan showing normal biodistribution of ^131^I with expected intense gastric uptake at the site of administration. Two focal areas of uptake are noted in the neck, raising concern for residual thyroid tissue or persistent disease. **(B)** Image demonstrating the exchange of the existing bolus PEG tube for a MIC-KEY low-profile gastrostomy feeding tube. **(C)** Post-therapy whole-body scan demonstrating a distribution pattern similar to the pre-therapy scan. A star artifact is observed in the neck, corresponding to intense activity from residual thyroid tissue.

A 37-year-old woman presented with a palpable neck mass. Ultrasound examination revealed a suspicious cystic lesion measuring 5.5 cm in the left thyroid lobe. The patient initially underwent fine-needle aspiration cytology (FNAC), which demonstrated scant follicular cells and was deemed non-diagnostic. Subsequently, the patient underwent a left thyroid lobectomy and isthmectomy with left-sided modified neck dissection due to suspicious imaging features. Histopathologic analysis confirmed multifocal papillary thyroid carcinoma (PTC), conventional subtype, with the largest focus measuring 5.2 cm and harboring a BRAF V600E mutation. The tumor was confined to the thyroid gland, with no intrathyroidal psammoma bodies or angioinvasion identified. Three metastatic cervical lymph nodes were detected in the left level VI and VII compartments, the largest of which measured 0.4 cm. A complete thyroidectomy was subsequently performed at a different institution. Final staging was Stage I (T3aN1aM0) according to the 8th edition of the AJCC criteria. As the patient was referred from an outside institution, detailed documentation of the preoperative diagnostic workup and clinical symptoms was not fully available. The clinical history reported in this manuscript is based on patient-reported information and limited outside medical records and was documented in the endocrinology consultation notes uploaded to our institutional electronic medical record at the time of referral. This represents a limitation of the available medical documentation rather than a limitation of the study itself. The patient’s completion thyroidectomy was unfortunately complicated by supraglottic stenosis and airway injury during a concurrent robotic lingual tonsillectomy, resulting in persistent dysphagia and dysphonia. A fluoroscopic video swallow study with speech was performed to assess for aspiration risk. The study showed an impaired pharyngeal phase, with aspiration observed when the patient swallowed level 2 mildly thick nectar liquids; a cough response was noted. Penetration to the vocal cords was observed with large volumes of applesauce and level 3 moderately thick honey liquids administered via a cup; these were cleared with a strong cough reflex. Additional findings included decreased hyolaryngeal excursion, complete epiglottic inversion, suspected decreased base-of-tongue strength as evidenced by vallecular residue post-deglutition, and suspected decreased pharyngeal strength as evidenced by residue post-deglutition. Based on her high aspiration risk, a percutaneous endoscopic gastrostomy (PEG) tube was placed to support long-term enteral nutrition. The patient was referred by the endocrinology service to the nuclear medicine department for pre-therapy imaging and evaluation for potential radioactive iodine (RAI) therapy (day 0). In preparation for the pre-therapy scan, the patient was placed on a two-week low-iodine diet and initiated on a 2-day thyrotropin alfa (Thyrogen; Genzyme Corp.) stimulation protocol. She presented to the nuclear medicine department on day 2 of the protocol, having received 0.9 mg of intramuscular thyrotropin alfa intramuscularly on days 1 and 2. An A Thyroid-Stimulating Hormone (ATSH) level of 109.7 μIU/mL confirmed adequate stimulation. Her baseline TSH level prior to thyrotropin alfa administration was 0.13 μIU/mL, peaking at 109.7 μIU/mL on day 2 following two intramuscular doses of thyrotropin alfa. The patient’s stimulated thyroglobulin level was measured on the same day (day 2) and increased to 0.9 ng/mL from 0.15 ng/mL, with thyroglobulin antibody levels remaining unchanged at <0.9 IU/mL. On the same day (day 2), the patient received a diagnostic dose of 81 MBq (2.2 mCi) of 131I Sodium Iodide Solution USP manufactured by Jubilant Radiopharam Jubilant Radiopharma, Montréal, Québec, Canada via her original PEG tube. The dose was carefully administered using a 10-cc syringe, ensuring no leakage or backflow. Whole-body anterior and posterior imaging, along with SPECT/CT of the neck, was performed 18 h post-administration (day 3, Figure 1A). The scan showed focal uptake in the thyroid bed, consistent with residual thyroid tissue, without evidence of abnormal cervical adenopathy or distant radioiodine uptake. Given the complexity of the patient’s case, including significant dysphagia, airway compromise, and a high risk of aspiration that precluded safe oral ^131^I capsule administration, a multidisciplinary team comprising endocrinology, nuclear medicine, radiopharmacy, radiation safety, and gastroenterology determined that a liquid formulation would be a safer alternative. However, the patient was unable to ingest the liquid ^131^I formulation due to severe swallowing difficulty. Following multidisciplinary consultation involving gastroenterology, nuclear medicine, nuclear pharmacy, radiation safety, and endocrinology, alternative administration options were considered (day 4).

1. Administration of liquid ^131^I via the existing bolus PEG tube.
2. Placement of nasogastric (NG) or nasojejunal (NJ) tube with advancement into the proximal small bowel, followed by delivery of liquid or capsule ^131^I.
3. Administration of Capsule ^131^I via endoscopy.

The primary concern with option 1 was the potential hazards associated with backflow of liquid ^131^I at therapeutic doses through the fixed external tubing or around the gastrostomy tube site. A gastrojejunal extension tube was considered, but this would require either fluoroscopic or endoscopic placement into the proximal small bowel with the risk of spontaneous migration back into the stomach at any point (a common and unpredictable occurrence). Option 2, using NG or NJ tube placement as suggested in prior reports (8, 9), was deemed unsuitable for this patient because of the risk of tube remigration into the stomach and retrograde leakage of radioactive liquid through the PEG tract, even after fluoroscopic or endoscopic confirmation of post-pyloric positioning. Endoscopic clipping of the tube within the duodenum was considered impractical, as the tube would need to be removed a few days later for bioassay, complicating accurate residual activity measurements. Administration of liquid ^131^I via a nasogastric tube was further avoided due to the potential for unacceptably high radiation exposure to staff during administration, particularly because spillage or emesis at the time of tube removal cannot be reliably predicted or prevented (9). Option 3: Capsule ^131^I administration via endoscopy was also evaluated. Although direct endoscopic deposition of the ^131^I capsule into the stomach has been reported as safe and effective in patients with significant dysphagia (10), this strategy was excluded due to the patient’s difficult airway and increased anesthetic risk, which posed substantial challenges for safely performing endoscopy and administering the therapeutic ^131^I capsule. Furthermore, this approach carries significant logistical limitations, including the need for immediate post-procedure radiation safety surveys and the potential risk of endoscope contamination if radiation levels exceed permissible thresholds, all of which are issues that could disrupt workflow and procedural capacity within the endoscopy suite.

Following a multidisciplinary discussion and given the patient’s intermediate-to-high risk classification according to the most recent ATA risk stratification system, in conjunction with uptake in the thyroidectomy bed on pre-therapy imaging and the patient’s preference for definitive treatment, an adjuvant radioactive iodine (^131^I) ablative dose of approximately 30 mCi was recommended in accordance with published ATA guideline recommendations. After careful deliberation, the team elected to replace the existing bolus PEG tube (EndoViveTM Percutaneous Endoscopic gastrostomy Kit Boston Scientific, Marlborough, Massachusetts, USA. with a MIC-KEY* low-profile gastrostomy tube (size 20 Fr × 5 cm; Avanos Medical Inc. Alpharetta, Georgia USA) to optimize radiation safety in preparation for I^131^I therapy (Figure 1B). Exchange of the feeding tube was readily feasible, given the tract was mature (i.e., >4 weeks old) and without the need for anesthesia/sedation. Compared to traditional PEG tubes with fixed extension tubing, MIC-KEY tubes offer a button-type, shorter external profile (with detachable external extension tubing), internal balloon retention, and a secure anti-reflux valve, enhancing seal integrity and minimizing leakage. These features improve patient comfort, reduce the risk of contamination, and facilitate safer delivery of radiopharmaceuticals, particularly therapeutic doses of I^131^ (11, 12). On day 4, TSH was rechecked before proceeding with therapy and found to have decreased to 44.09 μIU/mL, still sufficiently elevated to support effective treatment. Multiple TSH checks were necessary due to the complex coordination and accommodations required across several multidisciplinary teams. This approach ensured that the patient remained adequately stimulated for effective radioiodine therapy. A therapeutic dose of 1,110 MBq (30 mCi) of I^131^ Sodium Iodide Solution USP manufactured by Jubilant Radiopharam Jubilant Radiopharma, Montréal, Québec, Canada) Was prepared by the radiopharmacist in a 30-mL solution, diluted with distilled water, and dispensed in a 30-cc syringe vial with a screw cap. The solution was administered via an extension set connected to the MIC-KEY gastrostomy tube. A saline flush was performed beforehand to confirm tube patency and the absence of leakage. The dose was injected slowly by the nuclear medicine radiologist using appropriate shielding and in strict adherence to radiation safety protocols. Following administration, the line was flushed three times with 60 mL of distilled water to minimize residual activity and ensure complete delivery of the radioactive dose.

The patient remained under observation for 2 h to monitor for proper gastric emptying and potential adverse effects such as nausea or vomiting. Radiation safety measures, including Geiger counter surveys and wipe tests, confirmed no contamination. A thyroid bioassay was conducted 2 days post-treatment. Residual activity in the tubing and syringe was measured at 75,110 Bq (2.03 μCi), confirming safe and effective administration. After accounting for this residual activity, the actual dose delivered was calculated to be 1139.6 MBq (30.8 mCi). A follow-up whole-body scan performed 5 days post-treatment (day 9) demonstrated expected biodistribution, with intense uptake localized to the stomach at the administration site (Figure 1C). In addition, on the same day, the MIC-KEY PEG tube was replaced, and residual radioactivity was measured at 22,200 Bq (0.6 μCi). The patient was followed for 6 months, during which no delayed adverse effects or evidence of recurrent disease were identified. Thyroglobulin levels remained undetectable throughout follow-up, and she reported no nausea or treatment-related symptoms (see Table 1).

**Table 1:** Timeline of patient treatment.

Day	Description of event
Day 0	The patient was referred to the Nuclear Medicine clinic for further evaluation and consultation in preparation for RAI. Patient’s TSH baseline was checked before thyrotropin alfa stimulation protocol.
Day 1	The patient received the first dose of thyrotropin alfa injection.
Day 2	The patient received the second dose of thyrotropin alfa injection. The patient’s thyroglobulin tumor markers and antibody levels were checked, and TSH levels were rechecked following the thyrotropin alfa stimulation protocol. The patient received a diagnostic dose of 81 MBq (2.2 mCi) ^131^I administered via the PEG tube.
Day 3	Whole-body diagnostic scan images were acquired 18 h after ^131^I administration.
Day 4	A multidisciplinary consultation was conducted involving gastroenterology, radiation safety, nuclear medicine, radiopharmacy, and endocrinology. The patient’s TSH level was rechecked to confirm adequate stimulation in preparation for effective RAI. Exchange of existing bolus PEG tube with a MIC-KEY low-profile gastrostomy tube. Administration 1139.6 MBq (30.8 mCi) as therapeutic dose through MIC-KEY low-profile gastrostomy tube.
Day 9	Post-therapy images were obtained. Exchange of her MIC-KEY low-profile gastrostomy tube used for administration and bioassaying for any residual activity stuck in the tubing.

## Discussion

Radioiodine (^131^I) remains a cornerstone therapy for the management of DTC, with standardized treatment protocols widely adopted, as outlined by the Society of Nuclear Medicine and Molecular Imaging (SNMMI) and the European Association of Nuclear Medicine (EANM) (13). However, complex clinical scenarios can present unique challenges that necessitate deviation from conventional approaches. ^131^I is typically administered orally in capsule form, with the majority of the administered activity excreted via the urine. While oral administration is commonly used for most individuals, certain clinical scenarios, such as impaired swallowing, gastrointestinal absorption issues, or reliance on dialysis due to absent renal clearance, pose significant challenges to standard administration and elimination pathways. Although several prior studies have explored the challenges associated with ^131^I therapy and proposed alternative administration strategies, there remains a notable paucity of data in the medical literature regarding optimal management strategies for patients unable to swallow or ingest RAI, particularly those with severe dysphagia or high aspiration risk (8). These complex clinical scenarios necessitate individualized, innovative approaches tailored to each patient’s anatomical and functional limitations. Successful outcomes in such cases rely heavily on close coordination among multidisciplinary teams, including nuclear medicine, endocrinology, gastroenterology, anesthesia, radiation safety, and radiopharmacy. Equally critical is strict adherence to radiation safety protocols to ensure therapy is both effective and safe.

Although oral administration of ^131^I capsule is generally safe, as demonstrated by Bekier et al. with gastric radiation exposure remaining below harmful thresholds (14), patients with significant swallowing difficulties are at an increased risk of aspiration and administration errors (8). Few studies have addressed the challenges of ^131^I administration and proposed alternative strategies (8, 10, 15). In addition to swallowing difficulties. Halpern et al. (16) suggested that the formulation of iodine–gelatin complexes in the gastrointestinal tract may reduce mucosal exposure and radiation burden to the gastrointestinal tract by encapsulating doses in iodine–gelatin complexes or switching to liquid preparations. These adaptations aim to maintain therapeutic efficacy while minimizing exposure and contamination risks (16). In select cases, intravenous (IV) administration of ^131^I has been reported, primarily under special regulatory exceptions. This route may be beneficial for patients with severe gastroparesis or malabsorption syndromes, as it bypasses the gastrointestinal tract entirely. However, IV delivery presents its own set of challenges, including increased radiation exposure during preparation and injection, higher rates of nausea, and the absence of FDA approval or standardized dosimetry protocols. As such, IV administration remains off-label and is reserved for rare, highly selected cases (8, 17).

The final strategy considered to mitigate administration risks was direct endoscopic delivery of the ^131^I capsule into the stomach, an approach that reduces the potential for spillage and ensures accurate dosing (10). Endoscopic placement techniques were originally developed for capsule endoscopy in patients with dysphagia, and Shields and Johnson (10) adapted this concept to enable therapeutic ^131^I capsule administration in individuals unable to swallow safely. However, despite its theoretical safety advantages, endoscopic capsule delivery presents significant logistical and practical limitations. The procedure is invasive, often requires general anesthesia, and carries additional risks for medically complex patients. It may also disrupt workflow and raise important concerns regarding potential radiation contamination of endoscopic equipment, particularly in centers without established post-procedural decontamination protocols. In such situations, alternative enteric routes, such as administration through modified or low-profile gastrostomy tubes, including a MIC-KEY low-profile device, can offer a safe and effective method for ^131^I delivery when performed within a coordinated multidisciplinary framework. Because the therapeutic agent ultimately reaches the stomach, identical to standard oral administration, the expected therapeutic efficacy and long-term safety profile are comparable to conventional capsule swallowing. Compared with traditional PEG tubes that incorporate fixed external extension tubing, MIC-KEY low-profile gastrostomy tubes offer several safety and practical advantages. These devices are designed as a button-type system with a shorter external profile and detachable extension tubing, reducing external tubing length when not in use. This design improves patient comfort, minimizes accidental traction or dislodgement, and decreases the risk of contamination or leakage during administration. The internal balloon retention mechanism provides a secure intragastric seal, while the integrated anti-reflux valve further limits backflow and leakage at the stoma site. These features are particularly advantageous in the context of radioactive iodine (^131^I) therapy, where minimizing radiopharmaceutical spillage is critical for patient safety, radiation protection, and environmental contamination control. The secure seal and controlled delivery mechanism of the MIC-KEY system facilitate safe administration of therapeutic doses of ^131^I in patients unable to tolerate oral intake, such as those with dysphagia or aspiration risk (18, 19). Currently, there are limited published data directly comparing these devices for the administration of therapeutic radiopharmaceuticals.

In summary, our case highlights the feasibility and safety of liquid ^131^I administration via a MIC-KEY low-profile PEG tube in a patient with severe dysphagia. With proper technique and thorough post-administration flushing, residual radioactivity in the delivery tubing was minimized. Pre-administration leak testing and post-delivery measurements confirmed that the dose was effectively administered into the stomach, with negligible loss or contamination risk to staff or the patient. To our knowledge, no standardized guidelines currently exist for managing patients who are unable to tolerate oral or liquid ^131^I via traditional routes. This underscores the need for individualized treatment planning and the critical role of multidisciplinary collaboration. Additionally, it highlights the need for consensus-driven protocols and evidence-based strategies to ensure optimal RAI therapy in patients with impaired swallowing or limited gastrointestinal access.

## Data Availability

The original contributions presented in the study are included in the article/supplementary material; further inquiries can be directed to the corresponding author.
